# Adhesive Small Bowel Obstruction: A Review

**DOI:** 10.31729/jnma.8134

**Published:** 2023-04-30

**Authors:** Pradeep Ghimire, Shailesh Maharjan

**Affiliations:** 1 Department of Surgery, Manipal College of Medical Sciences, Fulbari, Pokhara, Nepal

**Keywords:** *diagnosis*, *laparotomy*, *prevention*, *surgery*

## Abstract

Adhesion is a leading cause of small bowel obstruction. Adhesive small bowel obstruction has significant challenges in diagnosis, treatment and prevention with considerable impact on morbidity and socioeconomic burden. Small bowel obstruction caused by adhesion or any other aetiology is clinically indistinguishable due to similar clinical presentation. Computed Tomography scans and water-soluble contrast studies are more specific in diagnosis and possess value in predicting the need for surgery. Surgical management is indicated only in complicated cases or failed conservative treatments with the majority resolving with non-operative management. However, there is no clear-cut consensus about the timing of operative intervention. Meticulous surgical practice is the keystone in preventing adhesion formation despite the availability of numerous pharmacological and surgical strategies. This review aims to update the current knowledge of the pathophysiology of adhesion formation, treatment options and various prevention modalities of adhesive small bowel obstruction.

## INTRODUCTION

Adhesion may be defined as abnormal inflammatory attachment between tissue surfaces.^1^ The most frequent cause of abdominal adhesion is previous abdominal surgery beginning to form within a few hours after the operation and responsible for 60% of subsequent intestinal obstructions.^2^ Despite improvements in healthcare, significant challenges regarding prevention, diagnosis and treatment with a huge influence on morbidity and socioeconomic burden exist. Although various pharmacological agents and surgical strategies have been developed, none have shown impressive effects to prevent it. The objective of this review is to update the present knowledge of the pathophysiology of adhesion formation, treatment options and various prevention modalities of Adhesive small bowel obstruction (ASBO).

## RISK FACTORS

The most important risk factor for abdominal adhesion formation is the type of surgery and the extent of peritoneal damage. Lower abdomen and pelvic surgeries have a higher risk of adhesion formation than upper abdominal surgeries.^3^ The median time of first operation for ASBO is 0.9 to 1.4 years after colorectal surgery, 1.8 years after hepatobiliary surgery, 2 years after an appendectomy, 4 years after gastric surgery and 7 years after gynaecological surgery.^4^ The incidence of ASBO has beenis open cholecystectomies vs 0.2% in laparoscopic; 15.6% in open total abdominal hysterectomies vs 0.0% in laparoscopic; and 23.9% in open adnexal operations vs 0.0% in laparoscopic with no statistical difference following laparoscopic or open appendectomies (1.4% vs 1.3%).^5^ The timing of the first presentation varies according to studies but in general 20% present within one month of surgery, 30% within one year, 25% in subsequent one to five years and the final 25% occur in five to twenty-five years.^6^ The relative risk of recurrence of ASBO increases with subsequent abdominal operations which are 1.6, 1.8 and 3.2 after one, two and three or more abdominal operations respectively.^5,7^

ASBO is a frequently encountered problem in general surgical practice and accounts for 20% of all surgical emergencies.^8^ Despite general improvements in healthcare, it still carries significant challenges regarding prevention, diagnosis and treatment with a huge influence on morbidity and socioeconomic burden. Most often there is confusion between small bowel obstructions (SBO) caused by adhesion and other etiologies with further causes a dilemma in proceeding with conservative management or surgical management. Although various pharmacological agents and surgical strategies have been developed to prevent the formation of adhesion, none have shown impressive effects. The lifetime risk of requiring hospital admission for adhesive small bowel obstruction (ASBO) following abdominal surgery is around four percent while requiring laparotomy is around two percent.^9^

## PATHOPHYSIOLOGY OF ADHESION FORMATION

### Increased Cellular Response to Tissue Damage

The visceral peritoneum is lined by a single layer of mesothelial cells which are loosely anchored to the basement membrane and are easily detachable on the slightest of trauma.^10^ Peritoneal healing (mesothelialization) differs from healing in skin, where reepithelialization occurs from the periphery inward. In the peritoneum, operative or traumatic defects are reperitonealized by the implantation of mesothelial cells in multiple areas of the defect.^11^ On peritoneal injury, the detached mesothelial cells express cell adhesion molecules and various chemotactic factors which cause a large influx of inflammatory cells, predominantly macrophages. Macrophages produce inflammatory cytokines like IL-1, IL-6 & TNF and antiinflammatory cytokines like IL-10 and Interferon-γ (INF-γ). IL-6 is a potent adhesiogenic whereas IL-1 and Tumor Necrosis Factor (TNF) are potent inducers of IL-6.^12^ A higher ratio of inflammatory cytokines to antiinflammatory cytokines promotes the formation of adhesion.^13^ These inflammatory responses are directly proportional to the extent of peritoneal damage.^14^

### Abnormal Fibrinolytic System

During peritoneal healing, there is a collection of fibrin-rich exudates in the injured area with simultaneous lysis. The fibrinolytic system degrades fibrin into fibrin degradation products by plasmin. The plasmin is activated from plasminogen by plasminogen activators (PA) such as tissue plasminogen activators (tPA) and Uroplasmin-like plasminogen activators (UPA) which can be inhibited by plasminogen activator inhibitors (PAI) such as PAI-1 and PAI-2. During injury to mesothelium, there is the deletion of the source of PA (tPA), as a result, the ratio of PAI to PA increases which leads to decreased fibrinolysis and stabilization of the fibrin matrix. Subsequently, fibroblasts proliferate and deposit extracellular matrix (ECM) including collagen which leads to the formation of adhesion.^10,15^

**Figure 1:**
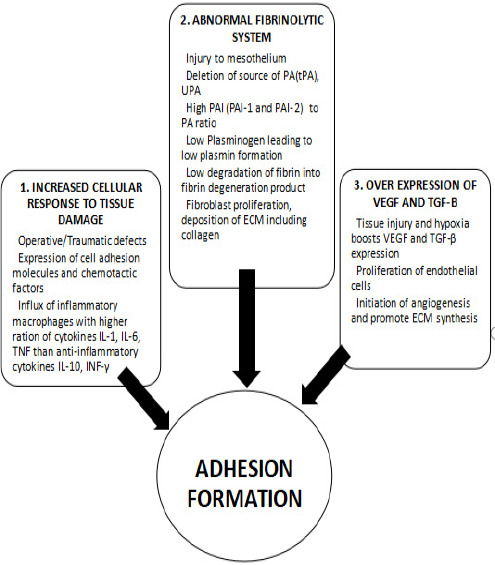
Pathogenesis of adhesion formation.

### Overexpression of Vascular Endothelial Growth Factor (VEGF) and Transforming Growth Factor (TGF-β)

Tissue injury and hypoxia promote the expression of vascular endothelial growth factor (VEGF) that boosts the proliferation of endothelial cells which initiate angiogenesis.^12^ Latent TGF-β is found in platelet, macrophages and wound fluid which is a potent stimulator of fibroblasts that promotes the synthesis of ECM. The over-expression of TGF and VEGF causes fibrosis and subsequent adhesion formation.^10^

## TYPES OF ADHESION

Adhesion is categorized mainly into fibrinous or fibrous. Fibrinous adhesion occurs during an early postoperative period which is avascular, flimsy and usually gets resolved completely. Fibrous adhesion is dense vascular adhesion in which the bowel gets attached to the peritoneum, omentum or other parts of the bowel that tends to persist and precipitate intestinal obstruction.^16^

## TYPES OF ACUTE SMALL BOWEL OBSTRUCTION (ASBO)

ASBO can be classified into complete or partial according to the degree of obstruction and early (<30 days after surgery) or late according to the timing of presentation.^17^ A study has classified ASBO into Type I adhesions which are formed at sites that did not have previous adhesion and Type II adhesions which are reformed adhesions at the sites that had previous adhesion. Type 1 is further classified into Type 1A which has previous operative procedures and Type 1B, in which there is no previous operative procedure at the site of adhesion. Type 2 is also classified into Type 2A which has no operative procedure other than adhesiolysis and Type 2B, in which other operative procedures are performed at the site of adhesion.^12^

## DIAGNOSIS

### Clinical Presentation

The patient of ASBO, like any other SBO, presents with complaints of abdominal pain, nausea, vomiting, abdominal distention and obstipation.^18^ Abdominal pain is the initial and most prominent symptom which occurs in paroxysms at four to five-minute intervals. Gilroy Bevan's triad of adhesive pain consists of (1) pain gets aggravated or relieved on change of posture, (2) pain in the region of the old abdominal scar and (3) tenderness elicited by pressure over the scar.^19^ Nausea and vomiting are more prominent with a higher obstruction while abdominal distention is prominent with distal obstruction. Obstipation is a later development.

### Physical Examination

On clinical examination, the patient may demonstrate severe signs of dehydration. The abdomen shows distention and scars from previous surgery may be noted if present. Early in the course of bowel obstruction, peristaltic waves may be visible particularly in thin patients along with hyperactive bowel sounds which later gradually disappear. Mild abdominal tenderness may be present.^20^ Severe tenderness, guarding and rigidity along with fever, tachycardia and leukocytosis are observed in the settings of strangulations and peritonitis.^21^ Hernias and intra-rectal masses can be excluded with groin and rectal examinations respectively.

## RADIOLOGICAL STUDIES

### Plain X-ray of Abdomen-Supine and Upright

Usually, plain radiographs of the abdomen are the first investigation of choice because of widespread availability and low cost. They have sensitivity, specificity, and accuracy from 79% to 83%, from 67% to 83%, and 64% to 82%, respectively to diagnose ASBO.^22^ The triad of dilated bowel loops (>3 cm in diameter), multiple air-fluid levels in upright film and paucity of air in the colon is suggestive of SBO.^23^ The inability to differentiate obstruction caused by adhesion from other aetiology and false negative findings in closed-loop obstruction are the major limitations of plain radiographs.

### CT Scan

CT scan is not routinely performed because of their cost and availability. It is usually done when clinical history, physical examination, and plain film are not conclusive. It is 90% to 94% sensitive, 96% specific and 95% accurate in diagnosing ASBO.^19^ Discrete transition zone with dilatation of bowel proximally and decompression distally, intraluminal contrast not passing beyond the transitional zone and little gas or fluid in the colon are suggestive features of small bowel obstruction in CT scan.^24^ Clustered bowel loops, beak sign, fat notch sign and small-bowel faeces sign are some of the specific findings observed in adhesive small bowel obstruction.^25^

### Ultrasound

USG has limited value in diagnosing ASBO. It is highly operator-dependent, air may obscure the underlying findings and be difficult to perform in obese patients. But maybe particularly important in pregnancy and to rule out other diagnoses.^11^

### Water-Soluble Contrast (Gastrografin) Study (WSCS)

A hyperosmolar water-soluble contrast medium, meglumine amidotrizoate (gastrografin) is valuable in patients undergoing initial non-operative management. It has both diagnostic and therapeutic value. It activates the movement of water into the small bowel lumen, decreases oedema of the bowel wall and enhances smooth muscle contractile activity which can generate effective peristalsis and overcome the obstruction.^26^ This study can be performed immediately at the time of admission or after an attempt at initial traditional conservative treatment. It can rule out complete ASBO and predict the need for surgery. Those who fail to show contrast in the colon within 24 hours usually need surgical intervention.^27^

### Enteroclysis

Small bowel enteroclysis is useful for the diagnosis of low-grade partial intermittent SBO. About 200 ml to 250 ml of barium followed by one to two litres of a solution of methylcellulose in water is instilled into the proximal jejunum via a long nasoenteric catheter and the movement is followed fluoroscopically.^28^ It can provide valuable information regarding the site, extent, severity and possible cause of obstruction.^29^ Disadvantages are slow transit of contrast material in the fluid-filled hypotonic small bowel, the need for gastroenteric intubation and the expertise to perform.

## LABORATORY TEST

Laboratory tests are not helpful in the actual diagnosis of ASBO but are important to assess the general condition of the patient. Serum electrolytes and hematocrit denote the degree of dehydration and adequacy of fluid resuscitation. Leucocyte counts, serum amylase, alkaline phosphatase, D-lactate, urea and creatinine level may be elevated in the settings of strangulation and ischemia.^30^

## MANAGEMENT

### Non-operative management

Partial ASBO and without signs of strangulation or peritonitis can safely undergo the initial trial of non-operative management with success in about 60% to 85% of patients.^31^ Conservative treatment involves nasogastric decompression, fluid and electrolyte correction and clinical observation. Urinary Catheterization with urine output monitoring, serial electrolyte measurement, hematocrit and WBC count is done. The use of broad-spectrum antibiotics is advocated by some with concerns about bacterial translocation.^32^ The non-operative management can be prolonged for up to 72 hours in absence of signs of strangulation or peritonitis.^27^ The onset of fever and tachycardia, leukocytosis >15000/mm^3^, elevated lactate level, metabolic acidosis, continuous severe abdominal pain (VAS>4) and abdominal guarding along with abdominal CT findings of pneumatosis intestinalis, portal venous gas, lack of small bowel faeces sign, devascularized bowel indicate strangulation and mandate immediate surgical intervention.^27,33^ Absence of contrast in the colon after 24 hours of gastrografin study and failure of conservative management for 72 hours also dictates the need for operative management.^34^

### Surgical Management: Open vs Laparoscopic

The presence of complications and failure of NOM mandates surgical intervention. The surgery itself can cause new adhesion formation with approximately 10% to 30% requiring another laparotomy for recurrent bowel obstruction.^35^ The open approach is generally preferred for strangulating ASBO. However, inadvertent enterotomy is noted in about 20% with open approach vs 1% to 100% with laparoscopic.^36^

Laparoscopic lysis of adhesion is feasible only in uncomplicated ASBOs and in selected patients.^37^ The factors associated with successful laparoscopic management are hemodynamically stability, absence of peritonitis or severe intra abdominal sepsis, proximal SBO, the first episode, absence of severe abdominal distension, low peritoneal Adhesion Index score in <3 abdominal quadrants, single band adhesion, experience and skills of the surgeon.^27^ Laparoscopic lysis of adhesion has potential advantages of less postoperative pain, faster return of intestinal function, shorter hospital stay and reduced recovery time, and decreased wound complications.^38,39^

## PREVENTION OF ADHESION

AThe goal of adhesion prevention is to abolish or reduce the incidence, severity, extent and complications of adhesion while preserving normal healing. Various agents and strategies have been proposed in preventing postoperative adhesion formation.

### General Measures

Opting for a minimally invasive approach whenever feasible reduces peritoneal damage thereby minimising adhesion formation.^27^ Strict adherence to meticulous surgical technique has been advocated for many years as a means to reduce adhesion formation. Minimization of peritoneal foreign body exposure (e.g. limited use of suture material, elimination of powder from gloves), careful and gentle tissue handling, limited use of cautery and retractors and sharp dissections whenever feasible, ensuring meticulous hemostasis while avoiding desiccation and ischemia, administering prophylaxis against infection, copious irrigation with 8 to 10 litres of saline to remove clots, prevention of spillage of contents like bile, faeces and covering anastomosis and raw peritoneal surfaces are some of the measures described and advocated.^13^

## PHARMACOLOGICAL AGENTS

Numerous pharmacological agents have been studied extensively in an attempt to prevent post-operative adhesion formation. The majority of the agents are limited to animal models but some noteworthy agents with promising results are:

### Antifibrotic Agents

Agents like citrate, heparin and dicumoral decrease the deposition of fibrin, which is necessary for adhesion formation.^13^ Halofuginone, an inhibitor type I collagen prevent the formation of permanent fibrous adhesion by decreasing collagen deposition in the fibrin matrix.^40^ These agents are effective in reducing post-operative adhesion in animal studies only.

### Anti-inflammatory Drugs

The effects of non-selective cyclooxygenase inhibitors like indomethacin, and nimesulide and selective cyclooxygenase inhibitors like celecoxib and rofecoxib have been studied in murine models. These agents interfere with prostaglandin synthesis and decrease the initial inflammatory response, vascular permeability and inflammatory cytokines.^41^ Their effectiveness is equivocal or even deleterious with abdominal wounds failing to heal.^42^

### Fibrinolytic Agents

These agents promote fibrinolysis by enzymatic degradation of fibrin once fibrin is formed. Examples are streptokinase, urokinase and synthetic tissue plasminogen activators.^10^ Their use is limited because of the significant cost and the risk of haemorrhage.

### Lubricating Agents

Lubricin is a glycoprotein found in articular cartilage with antiadhesive properties. The use of recombinant human lubricin in rat models reduced both macroscopic and microscopic intra-abdominal adhesions with minimal side effects.^43^ Human studies are yet to be done. Dilute hyaluronic acid solutions can be used to precoat the serosal surface during surgery and thereby decreasing post-surgical damage and adhesion formation.^44^

### Barrier Agents

Barrier agents separate injured peritoneal surfaces mechanically during the time of the post-operative healing period. They can be considered promising groups of agents in decreasing surgically induced adhesion. They exist in the form of a solution, membrane or gel. Icodextrin 4% solution, acts as an osmotic agent that retains fuid within the peritoneal cavity for 3 to 4 days and prevents contact between organs using hydro floatation.^45^ Modified oxidised regenerated cellulose (Interceed) is the first barrier to demonstrate efficacy in humans which forms a gel and physically separates tissue layers during tissue healing. Hyaluronan and carboxymethyl cellulose-based bioresorbable membrane (Seprafilm) are used as a barrier to keep the intestines separated until the mesothelial lining is restored.^46^ It has shown only moderate efficacy because of short life in the peritoneal cavity with increased anastomotic leaks when wrapped around the newly created anastomosis and suture lines.^47^

## SURGICAL METHODS

Numerous surgical methods have been attempted to prevent adhesion formation. A simple procedure developed by T.B. Noble in 1937 known as Noble's plication, wherein adjacent coils of the small bowel are sutured together to create controlled and predictable adhesion to prevent recurrent adhesive intestinal obstruction.^48^ In Childs-Phillips mesenteric plication technique, the small intestinal mesentery is plicated to prevent crumpling of the bowel and adhesion formation.^49^ A procedure of intraluminal splinting with Baker's tube was developed as an alternative to extraluminal methods of Noble's plication and Child Philips mesenteric plication. A long stiff tube is inserted through jejunostomy or through the nasogastric route up to the entry into the large bowel, which acts as an intraluminal splint.^50^ Most of the proposed surgical methods are rarely performed these days and are only of historical importance.

## WAY FORWARD

All abdominal surgery possesses a risk for postoperative adhesion formation which increases with subsequent operations. The severity of peritoneal damage and the imbalance between fibrin deposition and degradation during healing are the major inciting factors for intra-abdominal adhesion formation. Plain abdominal X-rays can sufficiently diagnose small bowel obstruction but CT scans and water-soluble contrast studies are more specific for ASBO. In absence of signs of peritonitis or strangulation, the patients can safely undergo a trial of NOM for 48 to 72 hours. Open surgery is preferred for complicated cases while laparoscopic management is feasible in selected patients only. There are no consistent satisfactory results with various proposed adhesion-preventing agents and strategies and are limited to animal models only. There is a need for more advanced studies and human trials to develop effective agents and strategies that prevent adhesion without impairing the normal healing process. Meticulous surgical practice and the use of barrier agents are the only strategies currently competent to prevent adhesion formation and its subsequent complications.
